# Influences of Rearing Season, Host Plant, and Silkworm Species on Gut Bacterial Community

**DOI:** 10.3390/insects16010047

**Published:** 2025-01-06

**Authors:** Chang Chen, Yujuan Hao, Jiaqi Yang, Jingyu Zhang, Huan Wang, Yanqun Liu

**Affiliations:** College of Bioscience and Biotechnology, Shenyang Agricultural University, 120 Dongling Road, Shenyang 110866, China; 15698772709@163.com (C.C.); 15184029381@163.com (Y.H.); rosalind0128.957@gmail.com (J.Y.); mirror123zhang@163.com (J.Z.)

**Keywords:** gut bacterial community, rearing season, host plant, silkworm species, edible insects

## Abstract

The gut bacterial community plays many important roles in the production of nutrients and digestion. *Antheraea pernyi* and *A. yamamai* (Lepidoptera: Saturniidae) are two traditional sources of human food, as well as being silk-producing insects. In the present study, the influences of rearing season (spring and autumn), silkworm species (*A. pernyi* and *A. yamamai*), and host plant (*Quercus wutaishanica* and *Salix viminalis*) on gut microbiota diversity were tested using Illumina MiSeq technology. We found that the bacterial composition and diversity of larvae reared in the autumn are elevated compared to those of larvae from the spring. Silkworm species played an important role in the gut bacterial community. Host plants also affected the diversity of the intestinal flora of the insects: the diversity of the intestinal flora of *A. pernyi* reared using *S. viminalis* was higher than those reared using *Q. wutaishanica*. Our findings provide insights into the gut microbial environment in edible insects.

## Simple Summary

The diversity characteristics of the gut microbial community in silkworms are important for a comprehensive understanding of green and sustainable ecology and chemistry in the silkworm industry, as well as for the improvement of their economic traits. We used two economically important silkworms, namely Chinese and Japanese oak silkworms (*Antheraea pernyi* and *A. yamamai*), to analyze the effects of rearing season, host plant, and silkworm species on the diversity of the larval gut bacterial community. The results were helpful for understanding of the gut bacterial community in silkworms.

## 1. Introduction

Traditional edible insects, such as the Chinese oak silkworm *Antheraea pernyi* (Lepidoptera: Saturniidae) and the Japanese oak silkworm *A. yamamai*, have been supplementary food sources in recent years [1,2]. For coarse silk production and human food sources (larvae, pupae, and moths), Chinese oak silkworm is a semi-domesticated silkworm that has been used for about five hundred years in China [3,4]. Today, it is commercially cultivated mainly in China, India, Japan, and Korea. About 8 million kilograms of cocoons (inside pupae) is yielded in China per year. The polyphagous larvae can feed on the leaves of oaks (*Quercus* spp.) and the basket willow (*Salix viminalis*) [5]. The Japanese oak silkworm is a wild species of silkworm that produces a naturally green silk, distributed throughout China, Korea, Japan, and Russia; and also feeds on the leaves of oaks and basket willow, like *A. pernyi* [6]. This insect is a new biomaterial product in human health research, and its pupae have high nutritional value [7,8]. To date, the larvae of two insects have still been reared outdoors.

Nutrient absorption and utilization are directly linked to the yield and quality of silkworm production. The insect’s gut is an important place to digest food and absorb nutrients for growth and development. As an integral component of the invertebrate host, the gut microbiota play a key role in the maintenance of normal gut function, including nutrient absorption, nutrient utilization, defense against pathogens, transfer of signals, and improvement of immunity [9,10,11,12,13]. The relationship between microbial communities and the silkworm can affect the health and nutrient absorption of the host and has attracted increasing interest due to its ecological and economic importance [14,15]. Information on the intestinal bacterial community of this economic insect will contribute to improving health and nutrient absorption of silkworms [16,17]. Basic information on the intestinal bacterial community of *A. pernyi* has been reported, but little knowledge is available on the intestinal microbiota of *A. yamamai* [18]. It is known that the economic traits of silkworms are closely related to the rearing season and rearing plant [19]. *A. pernyi* larvae grew faster when they were fed on *S. viminalis* than on *Q. wutaishanica*. The economic traits are also different when silkworm is reared on different foods or in different seasons. Whether the rearing season influences bacterial communities in *A. pernyi* remains to be addressed to provide a strategy to improve the silkworm production industry.

In the present study, we characterized the intestinal bacterial communities of larvae from two economically important silkworm species (*A. pernyi* and *A. yamamai*), associated with two host species (*Q. wutaishanica* and *S. viminalis*) and two seasons (spring and autumn), using Illumina MiSeq technology in order to obtain information about the microbial communities associated with the two silkworms. Our results point to significant effects of the rearing season, host plant, and silkworm species on the diversity and structure of the midgut microbiota.

## 2. Materials and Methods

### 2.1. Insect Culture Conditions and Sample Preparation

The Silkworm Experimental Field with oak trees (*Q. wutaishanica*) or willow trees (*S. viminalis*) is located in Shenyang Agricultural University (N41°50′3.00″; E123°34′18.03″). The larvae of *A. pernyi* and *A. yamamai* were reared in this field. The experimental area where we planted *Q. wutaishanica* and *S. viminalis* was 13 m long and 6 m wide and shared feeding niches for the larvae of the two silkworms. Two generations of *A. pernyi* strain Yuda No. 1 were successfully reared at the Silkworm Experimental Field and were kindly provided by the Henan Sericultural Research Institute, Zhengzhou, China. *A. yamamai* is a univoltine (one generation per year) insect, and its eggs were kindly provided by Heilongjiang Sericultural Research Institute, Ha’erbin, China. All of *A. pernyi* and *A. yamamai* larvae were derived from the same mother female moth to eliminate maternal differences.

We chose to study fifth-instar larvae since they represent the last instar of the caterpillars of both silkworm species. *A. pernyi*, as a bivoline (two generations per year) insect, was used to study the influences of rearing season on gut bacterial community. The larvae were fed on the same plant species (*Q. wutaishanica* or *S. viminalis*) after hatching in the spring and autumn. We randomly selected the experimental silkworm larvae. The fifth-instar larvae before the gluttonous stage were surface-sterilized in 70% alcohol for 2 min, transferred onto clean tissue to dry in a laminar airflow cabinet for 3 min, and dissected under aseptic conditions. The midgut was dissected from the live larvae and was washed using sterile saline three times and transferred into a 1.5 mL sterile centrifuge tube. The samples were collected individually and stored at −70 °C until their use.

We named the collected samples according to the convention of AP/AY, with AP representing *A. pernyi* and AY representing *A. yamamai*. The host samples were referred to with QW/SV, with QW representing *Q. wutaishanica* and SV representing *S. viminalis*. The rearing seasons were referred to with A/S, with A representing autumn and S representing spring.

### 2.2. Total DNA Extraction and Purification

The experimental samples were transported in dry ice. DNA extraction, library preparation, and DNA sequencing were carried out by Majorbio BioTech Co., Ltd. (Shanghai, China). The sample (200 mg) from each silkworm larva was a replicate, and each treatment had 4 replicates. A DNA Kit (Z.E.N.A^®^ Soil DNA Kit, Omega Bio-tek, Norcross, GA, USA) was used to extract the total DNA. We used a NanoDrop 2000 UV-vis spectrophotometer (Thermo Scientific, Wilmington, NC, USA) to determine the final DNA concentration and purification. Agarose gel electrophoresis (1%) was used to check the DNA quality.

### 2.3. PCR Amplification and Sequencing

Based on the pre-test, the primer pair 338F (5′-ACTCC TACGG GAGGC AGCAG-3′) and 806R (5′-GGACT ACHVG GGTWT CTAAT-3′) was used to amplify the target, the V3-V4 region of the bacterial 16S rRNA genes [20]. A total of 20 μL of PCR reaction mixture, including template DNA (10 ng), 5×FastPfu Buffer (4 μL), 2.5 mM dNTPs (2 μL), 5 μM of each primer (0.8 μL), and FastPfu Polymerase (0.4 μL), was processed using the following procedure: 95 °C for 3 min, 29 cycles at 95 °C for 30 s, 55 °C for 30 s, 72 °C for 45 s, and 72 °C for 10 min. A PicoGreen fluorometer was used to purify, quantify, and homogenize the amplified products (~468 bp). According to the manufacturer’s instructions, the Illumina MiSeq PE300 platform was used to accomplish the PCR product sequencing.

### 2.4. Data Processing

The sequence data were analyzed on the free online Majorbio I-Sanger Cloud platform (https://cloud.majorbio.com, accessed on 20 May 2024). Trimmomatic was used to demultiplex and quality-filter the raw FASTQ files. And then, FLASH (http://sourceforge.net/projects/flashpage/, accessed on 20 May 2024) was used to merge the sequence data (read) according to the criteria as follows: (1) Over a 50 bp sliding window, reads at any site that received an average quality score < 20 were truncated. (2) The primers were exactly matched, 2-nucleotide mismatching was allowed, and reads were removed if they contain ambiguous bases. (3) Overlaps longer than 10 bp sequences were merged on the basis of their overlap sequence.

We deposited the 16S rRNA gene sequences obtained in this study into SRA (Sequence Read Archive) under the accession numbers SRR9082165, SRR9082167, and SRR9082168 for *A. pernyi* fed on *Q. wutaishanica* in spring; SRR9093007, SRR9093009, and SRR9093011 for *A. pernyi* fed on *Q. wutaishanica* in autumn; SRR9093003, SRR9093005, SRR9093006, and SRR9093008 for *A. pernyi* fed on *S. viminalis* in autumn; and SRR9093001, SRR9093002, and SRR9093004 for *A. yamamai* fed on *S. viminalis* in autumn.

Operational Taxonomic Units (OTUs) with a 97% similarity cutoff were clustered using UPARSE (version 7.1 http://drive5.com/uparse/, accessed on 20 May 2024). UCHIME T was used to identify and remove chimeric sequences [21]. The RDP Classifier algorithm (https://sourceforge.net/projects/rdp-classifier/, accessed on 20 May 2024) was used to analyze the taxonomy of each 16S rRNA gene sequence against the Silva (SSU123) 16S rRNA database using a confidence threshold of 70% [22]. Rarefaction curves were designed to measure species richness and evenness in a sample. We used rarefaction curves, specifically for richness rarefaction and the Shannon index, to estimate the sampling completeness. The rarefaction curves for all samples were generated in different colors to represent different samples.

The alpha and beta diversity indices were evaluated using QIIME2. Alpha diversity is an important feature of a microbial community’s structure, which can reflect the abundance and diversity of the microbial flora through the species diversity in a single sample. Mothur was used to calculate the alpha diversity index for different random samples. The Chao index, the Ace index, the Shannon index, and the Simpson index were used to analyze the richness and diversity of the intestinal microbiota [23,24,25,26]. The Chao index and the Ace index were used to estimate the total number of species. The Shannon and Simpson indices were used to estimate the microbial diversity in the sample.

The beta diversity analysis was carried out using QIIME 2 software to compare the species diversity between different samples. The beta diversity analysis was assessedbased on PCoA in this study. By sorting a series of eigenvalues and eigenvectors, the first several main eigenvalues were selected. Dimensionality reduction was performed by creating new uncorrelated variables which could maximize the differences between treatments. Bray–Curtis dissimilarity was used to calculate the distance between two treatments, and Anosim (analysis of similarities) was the statistical method mainly used to analyze the similarity between multi-dimensional data treatments.

### 2.5. Statistical Analysis

A bioinformatic analysis of the gut microbiota was carried out using the Majorbio Cloud platform (https://cloud.majorbio.com, accessed on 20 May 2024). Based on the OTU information, rarefaction curves and alpha diversity indices, including the observed OTUs, Chao1 richness, the Shannon index, and Good’s coverage, were calculated using Mothur v1.30.1. The similarity among the microbial communities in the different samples was determined through a principal coordinate analysis (PCoA) based on the Bray–Curtis dissimilarity using Vegan package v2.5-3. The PERMANOVA test was used to assess the percentage of variation explained by the treatment, along with its statistical significance, using Vegan package v2.5-3. The linear discriminant analysis (LDA) effect size (LEfSe) (http://huttenhower.sph.harvard.edu/LEfSe, accessed on 20 May 2024) was calculated to identify significantly abundant taxa (phylum to genera) of bacteria among the different groups (LDA score > 2, *p* < 0.05). Student’s T-test was used to compare the differencebetween two treatments. All comparisons were considered significant at *p* < 0.05.

## 3. Results

### 3.1. General Analyses of the Dataset

**Figure 1:**
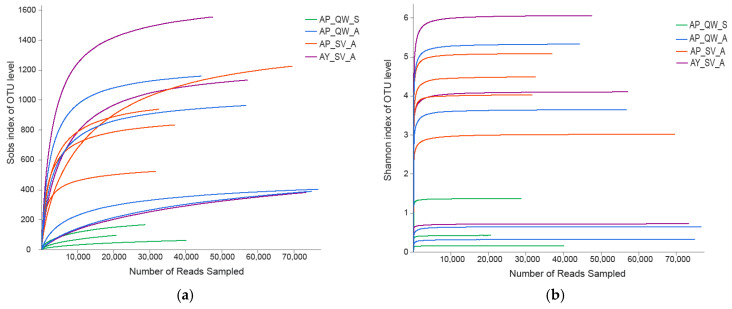
Richness rarefaction and Shannon index analysis of the silkworm samples used. (**a**) Rarefaction curves of OTUs clustered at a 97% sequence identity across samples. (**b**) Rarefaction curves of the Shannon index according to OTUs.

Using Illumina MiSeq sequencing of 16S rRNA genes, the present study yielded a total of 30,039, 65,251, 45,139, and 62,263 valid reads per sample for AP_QW_S, AP_ QW_A, AP_SV_A, and AY_SV_A, respectively, after the removal of low-quality reads (Table 1). In this study, the average length of each read was 428~450 bp. Valid reads exceeding 94.34% could be assigned to the genus level. Sequencing data were used to evaluate the diversity and richness of the intestinal bacteria (Figure 1). The rarefaction curves provided insight into how the sequencing depth affected the ability to fully capture a sample’s diversity. The sequencing depth was enough to uncover most of the biodiversity in the larval intestine according to the plateaued richness rarefaction curves for individual samples. Through the Shannon index, the bacterial diversity was estimated. The Shannon rarefaction curves tended to plateau, indicating that the bacterial diversity varied in the different samples. To minimize the effects of sequencing depth on the alpha and beta diversity measures, the number of 16S rRNA gene sequences from each sample was rarefied to 20,000, which still yielded an average Good’s coverage of 99.09%, respectively. The PCoA plots were described as showing sample clustering based on the microbiota diversity. Statistical metrics (R^2^ = 0.4449; *p* = 0.007) were provided to quantify the separation among groups.

### 3.2. The Effect of Silkworm Species on the Gut Microbiota

In this experiment, larvae of *A. pernyi* (AP_SV_A) and *A. yamamai* (AY_SV_A) were mixed together to feed on *S. viminalis*, thus providing us with the chance to assess the effect of silkworm species on the gut microbiota. Two silkworm larvae were reared outdoors on the same plant species (*Q. wutaishanica* or *S. viminalis*) after hatching. Unfortunately, we did not obtain enough *A. yamamai* larvae reared on *Q. wutaishanica* due to pests, diseases, and bird feeding. In addition, as food for *A. pernyi* and *A. yamamai*, *S. viminalis* grew faster and was managed more easily than *Q. wutaishanica*. The larvae of *A. pernyi* grew faster when they were fed on *S. viminalis* than on *Q. wutaishanica*. Therefore, we used *S. viminalis* as the food to study the influences of the silkworm species on the gut bacterial community.

**Figure 2:**
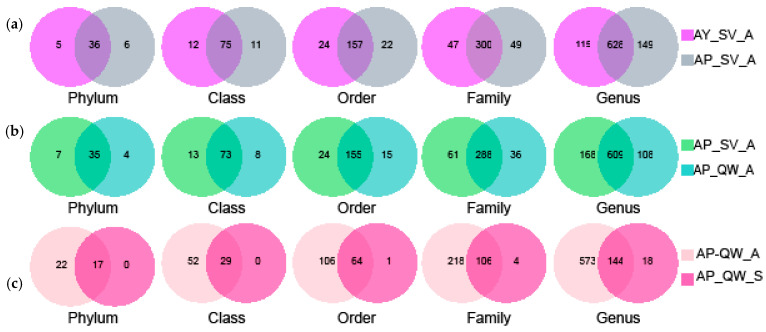
Shared bacterial types at different classification levels among samples. (**a**) Venn analysis of the midgut of *A. pernyi* (AY_SV_A) and *A. yamamai* (AY_SV_A) larvae fed on *S. viminalis*. (**b**) Venn analysis of *A. pernyi* larvae fed on *Q. wutaishanica* (AP_QW_A) and *S. viminalis* (AP_SV_A). (**c**) Venn analysis of *A. pernyi* larvae reared in spring (AP_QW_S) and autumn (AP_QW_A).

**Figure 3:**
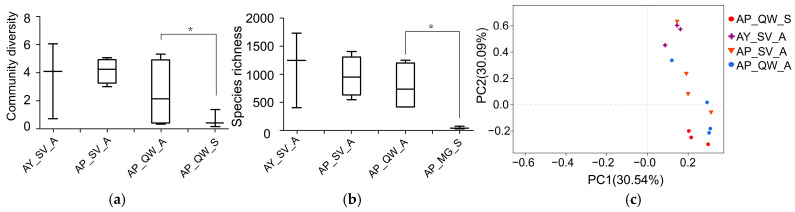
Bacterial community dynamics among samples. (**a**) Boxplot of species richness (number of OTUs). (**b**) Boxplot of community diversity measured using the Shannon index. (**c**) PCoA plot showing the variation in community structure. Principal components (PCs) 1 and 2 represented 30.54% and 30.09% of the variance, respectively. * indicates signifcant differences (*p* < 0.05).

The OTU representative reads for *A. pernyi* (values for *A. yamamai* are shown in parentheses) were assigned to the different levels of taxonomical classification as follows: 42 (41) phyla, 86 (87) classes, 179 (181) orders, 349 (347) families, 777 (743) genera, and 1281 (1274) OTUs. A Venn analysis showed that 36 phyla, 75 classes, 157 orders, 300 families, and 688 genera were common between the two silkworm species (Figure 2a). In terms of the estimated alpha diversity of the bacteria for *A. pernyi* (Table 2), the Chao indices, Ace indices, and Simpson indices were higher for *A. yamamai* than those for *A. pernyi*, but no certain trend was found for the Shannon indices between *A. yamamai* and *A. pernyi* (Table 2). The boxplot of species richness (number of OTUs) and the community diversity measured using the Shannon index indicated no significant differences between *A. yamamai* and *A. pernyi* (Figure 3a,b). These results indicated a silkworm-specific characteristic in terms of the bacterial composition at the level of different classification orders (Figure 2a), although they exhibited similar bacterial species richness and community diversity (Figure 3a,b). The PCoA also suggested a silkworm-specific impact on microbiota diversity (Figure 3c).

**Figure 4:**
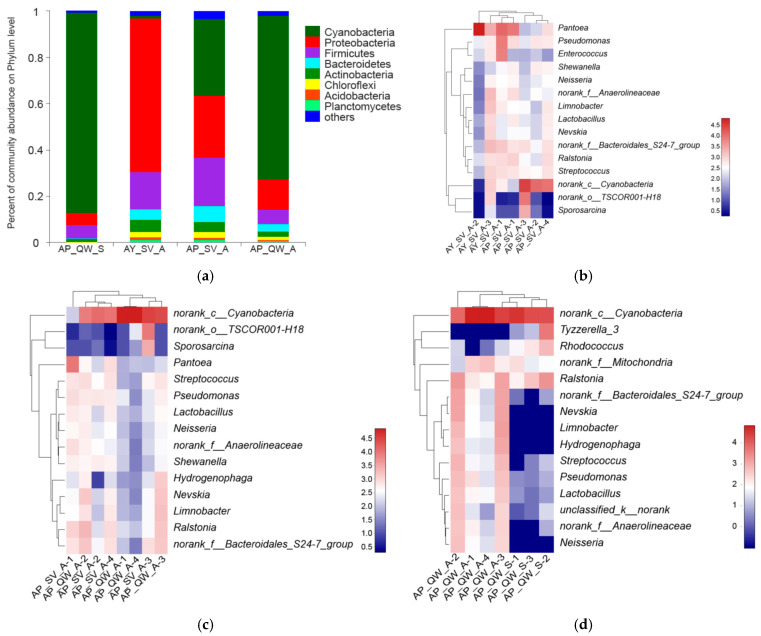
Comparison of the bacterial community among the different samples. (**a**) The dominant phyla percentage for AY_SV_A (n = 3), AP_SV_A (n = 4), AP_QW_A (n = 4), and AP_QW_S (n = 3). Bacteria with an abundance less than 1% are classified as others. (**b**–**d**) Heatmap of bacterial communities based on the average value of the top 15 dominant genera for AY_SV_A and AP_SV_A, AP_QW_A and AP_SV_A, and AP_QW_A (autumn) and AP_ QW_S (spring), respectively. Rows and columns represent the samples and the dominant genera, respectively. A deeper red color indicates a closer distance between the samples, and a deeper blue color indicates a larger distance between the samples.

Eight major bacterial phyla were identified in both groups, and Proteobacteria, Firmicutes, Cyanobacteria, Bacteroidetes, and Actinobacteria were the five most dominant phyla, accounting for 90~92% (Figure 4a). The proportion of Firmicutes between them was similar (17.94% vs. 18.01%). The abundance of Proteobacteria and Cyanobacteria in AY_ SV_A was 61.76% and 1.29%, whereas it was 30.18% and 30.61% in AP_SV_A, indicating that the abundance of Proteobacteria and Cyanobacteria differed significantly between them (df = 5, t = 23.11, and *p* = 0.000 in Proteobacteria; df = 5, t = −27.15, and *p* = 0.000 in Cyanobacteria).

At the genus level, seven dominant genera were common between them (Figure 4b), including Pantoea, nonrank_c_Cyanobacteria, Pseudomonas, nonrank_c_Bacteroidales_S24-7_group, Ralstonia, Streptococcus, and non-rank_f_Anaerolineaceae. However, Pantoea and nonrank_c_Cyanobacteria were significantly different between the two silkworm species. Pantoea was found at 42.16% in AY_SV_A and 7.49% in AP_ SV_A (df = 5; t = 25.38; *p* = 0.000), and nonrank_c_Cyanobacteria was found at 1.24% in AY_SV_A and 30.49% in AP_SV_A (df = 5; t = −19.15; *p* = 0.000).

### 3.3. The Effect of the Host Plants on the Gut Microbiota

The polyphagous larvae of *A. pernyi* can feed on the leaves of *Q. wutaishanica* (AP_QW_A) and *S. viminalis* (AP_SV_A), and the two host plants share microhabitats, thus providing us with a good chance to assess the effect of the two host species on the gut microbiota of this economical insect. The Chao indices, Shannon indices, and Ace indices were higher in the larvae feeding on *S. viminalis* than those feeding on *Q. wutaishanica*, but no given trend was found for the Simpson indices (Table 2). The boxplot of species richness and community diversity measured indicated no significant difference between the larvae feeding on *S. viminalis* and *Q. wutaishanica* (Figure 3a,b). The overall analysis indicated a host-specific characteristic in the bacterial composition at the level of different classification orders (Figure 2b), with relatively higher bacterial species richness and community diversity in AP_SV_A than those in AP_QW_A (Figure 3a,b). The PCoA did cluster these individuals separately (Figure 3c).

The phyla Bacteroidetes, Actinobacteria, Chloroflex, and Acidobacteria exhibited no significant differences between the two groups (Figure 4a). However, Cyanobacteria was more abundant in AP_QW_A (70.92%) than AP_SV_A (33.43%; df = 6; t = 15.75; *p* = 0.000), whereas Proteobacteria and Firmicutes were less abundant in AP_QW_A (13.08% and 6.14%) than AP_SV_A (26.64%, df = 6, t = −7.83, and *p* = 0.000 and 20.95%, df = 6, t = −8.55, and *p* = 0.000).

A heatmap provides a comparison of the differences between the two groups at the genus level (Figure 4c). The dominant bacteria *norank_c_Cyanobacteria*, *Pseudomonas*, and *Ralstonia* were present in all samples. Specifically, *Cyanobacteria* increased from 30.49% in AP_SV_A to 64.10% in AP_QW_A (df = 6; t = 19.41; *p* = 0.000), whereas *Pantoea* decreased from 7.49% in AP_SV_A to 0.40% in AP_QW_A (df = 6; t = −8.52; *p* = 0.000).

### 3.4. Comparison of the Gut Microbiota Between Different Seasons

To address the issue of whether the rearing season influences silkworm bacterial communities, the bacterial community in the midgut of *A. pernyi* larvae reared in spring (from May to June; AP_QW_S) was compared to that of those reared in autumn (from August to September; AP_QW_A) in this study. All of the larvae used were fed on *Q. wutaishanica* after hatching. An analysis of the shared taxa indicated that 17 phyla, 29 classes, 64 orders, 106 families, and 144 genera were common between the two seasons, and nearly all of the bacterial species found in the spring larvae (AP_QW_S) were covered by those in the autumn larvae (AP_QW_A) (Figure 2c).

The bacterial species richness and community diversity of those reared in autumn were greater than in those reared in spring (Figure 3a). The estimated alpha diversity of the bacteria in *A. pernyi* reared in two seasons (Table 2) showed that their Chao indices, Shannon indices, and Ace indices were higher in autumn than in spring, but no certain trend was found for the Simpson indices (Table 2). The boxplot of species richness and community diversity measured indicated that the Chao indices (df = 5; t = 2.947; *p* = 0.032) and Ace indices (df = 5; t = 2.984; *p* = 0.031) were significantly higher in autumn than in spring, and there were no significant differences in the Shannon indices and the Simpson indices between the two seasons (Figure 3a,b). The PCoA did cluster their individuals separately (Figure 3b). The dominant phyla percentage (Figure 4a) and the heatmap (Figure 4d) also exhibit the differences at the phylum and genus levels between them. Note that *Tyzzerella_3* bacteria were dominant in spring but not in autumn.

## 4. Discussion

Many factors, such as strain, forages, developmental stage, gender, health status, living conditions, and insect species, can shape the silkworm’s gut microbial community [27,28,29,30]. In this study, our data from *A. pernyi* and *A. yamamai* associated with two host plants *Q. wutaishanica* and *S. viminalis* further highlighted silkworm- and host-plant-specific effects on the microbiota associations. We found that at the phylum level, Proteobacteria and Cyanobacteria dominated in *A. yamamai* and *A. pernyi*, respectively; the *Q. wutaishanica*-fed larvae were dominated by Cyanobacteria, while the *S. viminalis*-fed larvae were dominated by Proteobacteria and Firmicutes. Our study showed that the gut microbial community of different silkworm species reared on different host plants in different seasons was different.

The gut bacterial community in insects is seasonally variable [31]. The rearing season is closely related to the economic traits of silkworms [29]. This study analyzed the effect of rearing season on the diversity of the silkworm’s gut bacterial community. As important economic insects, characterization of the microbial community in their gut is essential for a comprehensive understanding of silkworm ecology, as well as for improvements in their economic traits [10,16]. Voltinism, the number of generations of an organism that occurs in a year, applies to insects and is particularly used in sericulture, with silkworm varieties varying in their voltinism. Silkworm varieties are then raised in different rearing seasons according to their voltinism. Given this, the economic traits of the Chinese oak silkworms are closely related to the rearing season [29], with bigger cocoons and pupae and an elevated nutritional composition for larvae reared in autumn than those reared in spring in bivoltine silkworm area. As expected, we found that the bacterial composition and diversity of *A. pernyi* reared in the autumn were elevated compared to those in the larvae in the spring, thus providing evidence that the rearing season has an impact on the diversity of the silkworm’s bacterial community. Previous studies have demonstrated that an insect’s microbiota is geographically stable considering the same host plant [32,33]. our results indicated that the rearing season played an important role in the insects’ microbiota in the same geographic location and host plant. An explanation may be that the season influences the endophytic bacterial communities in the leaves of the host plant [34]. Therefore, further studies dealing with the relationship between economic traits in silkworm and intestinal bacterial community using multiomics technology and bacterial function verification are worthy of being pursued in the future.

It is not surprising that insects’ gut microbiota can be influenced by their diet [35,36] due to the relatively simple morphology of the gut in lepidopteran larvae [37] and the lack of specific structures for harboring microbial symbionts. Insect species specificity in terms of gut microbial community has also been observed in *B. mori*, *Acronicta major,* and *Diaphania pyloalis*; all of these were mulberry-feeding relatives, just like those in our study. We inferred that the phylogeny of the insect species plays an important role in shaping the structure of the microbial community [2]. The host-plant-specific effect may be caused by the plant species influencing the endophytic bacterial communities in the leaves [34]. At the level of genus, the abundance of *Cyanobacteria* in AY_SV_A was 1.24%, which increased by 25 and 52 times in AP_SV_A and AP_QW_A, and the abundance of *Pantoea* in AP_QW_A was 0.40%, which increased by 19 and 105 times in AP_SV_A and AY_SV_A, respectively. The results indicated that *Cyanobacteria* was the resident flora in the epithelium of the midgut in *A pernyi* and the leaves of the host plant of *Q. wutaishanica*, and *Pantoea* was closely related to the leaves of the host plant *S. viminalis*.

In this study, we provided information about the gut microbial diversity of two wild silkworms, *A. pernyi* and *A. yamamai*. The two wild silkworms, which were reared in the field on oak leaves, were intimately associated with diverse populations of microbes that were dominated by five phyla of bacteria (Proteobacteria, Firmicutes, Cyanobacteria, Bacteroidetes, and Actinobacteria). These results have also been observed in the domesticated silkworm *B. mori* [2] and other insects [30]. Our results suggested that the microbiota composition of the midgut between the two silkworms belonging to the *Antheraea* genus had no dramatic changes at the phylum level. However, at the genus level, *A. pernyi* was dominated by *norank_c__Cyanobacteria* (86.22%), and *A. yamamai* was dominated by *Pantoea* (41.90%), which suggested a silkworm-specific effect on the microbiota associations.

*Ralstonia* bacteria, mostly related to the pathogenicity of plants, was found as the dominant bacterium in all of the surveyed samples. This bacterium not only has a broad host range across over 50 families but also causes serious harm worldwide [38,39]. The symbionts in insects are originally derived from free-living environmental microorganisms according to the results of previous studies [40]. We speculated that this bacterium was derived from the leaf surface of the host plants (*S. viminalis* and *Q. wutaishanica*). However, as the dominant bacterium, whether the *Ralstonia* bacteria affects silkworms’ health or productivity and the ecological role and potential implications of this bacteria are worth studying in future research.

## 5. Conclusions

The microbial communities that live inside the guts of insects have attracted increasing interest. In the present study, two importantly economical silkworms, namely the Chinese oak silkworm *A. pernyi* and the Japanese oak silkworm *A. yamamai*, were used to test the influences of rearing season, host plant, and silkworm species on the gut bacterial community using the Illumina MiSeq technology. We found that the rearing season of the larvae has an important impact on the gut’s microbiota diversity and the autumn larvae contain a more complex bacterial composition and greater diversity than the spring larvae. The diversity of the intestinal flora of *A. pernyi* reared on *S. viminalis* was higher than that in those reared on *Q. wutaishanica*. The findings presented here provide insights into the high complexity of the gut bacterial communities of insects.

## Figures and Tables

**Table 1:** Composition of intestinal bacterial communities of silkworm larvae.

Sample		Sequences	Phylum	Class	Order	Family	Genus	OTUs
AP_QW_S (spring)	1	40,091	9	14	27	39	50	54
	2	29,184	15	26	58	83	124	150
	3	20,842	14	19	41	63	76	89
	Total	90,117	17	29	65	110	162	206
AP_QW_A (autumn)	1	77,966	22	43	86	145	249	328
	2	47,763	31	67	144	264	528	819
	3	59,491	32	67	136	237	485	714
	4	75,785	20	38	87	145	258	338
	Total	261,005	39	81	170	324	717	1167
AP_SV_A (autumn)	1	39,036	31	67	135	235	449	626
	2	33,723	22	48	95	163	298	406
	3	73,121	27	62	138	263	580	892
	4	34,677	32	63	137	245	477	697
	Total	180,557	42	86	179	349	777	1281
AY_SV_A (autumn)	1	60,194	31	59	136	257	524	824
	2	74,352	18	35	76	138	242	322
	3	52,244	40	84	170	313	644	1065
	Total	186,790	41	87	181	347	743	1274

**Table 2:** Composition of diversity indices of silkworm larvae.

Sample		Chao	Shannon	Ace	Simpson
AP_QW_S (spring)	1	84.21	0.16	93.81	0.96
	2	213.00	1.37	205.73	0.45
	3	136.05	0.42	147.67	0.87
AP_QW_A (autumn)	1	458.22	0.65	449.11	0.86
	2	1206.67	5.33	1190.60	0.04
	3	1014.56	3.65	993.39	0.23
	4	509.99	0.33	524.79	0.93
AP_SV_A (autumn)	1	870.04	5.08	860.77	0.04
	2	555.39	4.02	547.38	0.16
	3	1345.86	3.01	1321.72	0.25
	4	985.58	4.48	975.85	0.12
AY_SV_A (autumn)	1	1192.98	4.10	1174.13	0.07
	2	528.04	0.72	546.26	0.68
	3	1631.35	6.06	1605.97	0.01

## Data Availability

The datasets presented in this study can be found in online repositories. The names of the repository/repositories and accession number(s) can be found in this article. Further enquiries may be directed to the corresponding author.
